# PD196 The Importance Of Promoting The Culture Of Health Technology Assessment Among Healthcare Professionals

**DOI:** 10.1017/S0266462324004185

**Published:** 2025-01-07

**Authors:** Laura Llinàs-Mallol, Berta Mestre-Lleixà, Rosa Maria Vivanco-Hidalgo

## Abstract

**Introduction:**

Last year we developed a survey focused on decision-making in health innovation within the health system of Catalonia. Evaluation of the survey results, coupled with the relationships we established with various members of the Catalan health ecosystem, has highlighted the need for informative activities on health technology assessment (HTA) that aim to bring HTA closer to healthcare professionals.

**Methods:**

We designed a two-hour training workshop divided into three thematic blocks and a group dynamic. The first block addressed HTA and the lifecycle of a health technology. The second and third blocks delved into the development of an HTA report, the role of healthcare professionals, and the formulation of a research question. During the workshops, we showcased a video and an infographic and distributed promotional bags to the attendees. A satisfaction survey was distributed at the conclusion of the workshop. We have conducted three editions of the workshop in a face-to-face format and one in a virtual format.

**Results:**

Of the 56 people who participated in the workshops, 48 responded to the satisfaction survey. The most frequent participants were directors, care coordinators, and health managers. The attendees rated the workshops with 4.6 out of five points. About 66.7 percent of attendees indicated that the workshop had met their training needs. Most attendees (79.2%) considered that the workshop had provided knowledge that they could apply to their daily tasks. Finally, when asked about topics of interest for future workshops, the participants voted for advanced HTA methodology, the development of HTA in healthcare centers, and the evaluation of digital health.

**Conclusions:**

The feedback from participants across all the workshops was very positive. The degree of satisfaction among attendees was high, with the main area for improvement being the need to develop new workshops to delve deeper into HTA. In 2024, we aim to design a training program consisting of HTA workshops that fulfil the requests of the attendees.

